# Tangsa and Wancho of North-East India Use Animals not only as Food and Medicine but also as Additional Cultural Attributes

**DOI:** 10.3390/foods9040528

**Published:** 2020-04-22

**Authors:** Salomi Jugli, Jharna Chakravorty, Victor Benno Meyer-Rochow

**Affiliations:** 1 Biochemical Nutrition Laboratory, Department of Zoology, Rajiv Gandhi University, Itanagar, Arunachal Pradesh 791112, India; galsalomi@gmail.com (S.J.); jharnargu@gmail.com (J.C.); 2 Department of Plant Medicals, Andong National University, Andong GB 36729, Korea; 3 Department of Ecology and Genetics, Oulu University, FIN 90140 Oulu, Finland

**Keywords:** common knowledge, traditional wisdom, North-East Indian tribals, Arunachal Pradesh, ethnobiology

## Abstract

Cultural and ritual uses of animals beyond those for food and medicine should not be dismissed if we wish to understand the pressure that wildlife is under. We documented such uses for the Tangsa and Wancho tribals of Eastern Arunachal Pradesh (India). Group discussions with assembled members of 10 accessible villages in each of the tribal areas were carried out in 2015 and 2016. Vernacular names of culturally important species were noted and details of hunting practices were recorded. The different uses of animals and their parts during rituals and festivals and their significance in decorations and adornments, in supernatural beliefs and in connection with tribal folklore (stories) are documented. Folklore helps us understand why some species are hunted and consumed while others for no apparent reason are killed or simply ignored. Similarities as well as differences between the two tribes were recorded and possible reasons for the differences are given. The roles that the government as well as the tribal leaders play to halt or slow down the erosion and gradual disappearance of traditions that define the two cultures without losing already rare and endangered species are highlighted.

## 1. Introduction

The Earth faces a biodiversity crisis, with current rates of species extinction that are between 1000 and 10,000 times higher than the background rate [1]. Conservation actions are a key part of responses to try to halt or minimize the loss of biodiversity [2]. Yet conflicts can emerge between the conservation of biodiversity and the uses of wildlife by people. It can be particularly difficult to resolve such conflicts where they relate to the traditional uses of wildlife by indigenous peoples, as the cultural practices of such peoples are often not well understood by conservationists or by government agencies tasked with conserving wildlife [3,4,5,6]. There is therefore a need to understand the uses of animals, including ritual and cultural uses beyond food and medicine, by tribes living in biodiversity hotspots. Such local knowledge, as well as being important cultural knowledge in its own right, is often overlooked by conservation.

Cultural knowledge is based on traditions. Frequently also referred to as common sense [7], cultural knowledge is the cumulative body of awareness and understanding of practices and beliefs held by local people. It involves adaptations to environmental circumstances, having stood the test of time, is handed down through generations by way of cultural transmission and is an important component of the cultural identity of tribal communities around the world [8,9,10,11,12,13].

Given the dearth of knowledge of the traditional roles that animals play beyond those as food and medicine [14,15], and in order to understand the pressure that wildlife is under, every bit of information still available must be welcomed. It is for this reason (and the fact that the first author of this paper is a Tangsa herself) that we chose to work with these two tribes of Arunachal Pradesh, a region of North-East India considered a global biodiversity hotspot [16]. Arunachal Pradesh is famous for its high levels of ecological, geographic and ethnic diversity, and the state is home to a variety of traditional communities with 26 major and 110 sub-tribes [6,17,18,19,20].

Tangsa and Wancho still have a close relationship with the animals of the region be it through the people’s awareness of the dangers that some species pose or through activities like trapping and hunting. This relationship has only been given some attention by Dutta [18] and Dutta and Bhattacharjya [20]. On the other hand, Tangsa history, community structure, social interactions, style of dwellings, etc., were covered by Rao [21] and Morang [22], while the marriage system of the Wancho was studied by Ralongham [23].

An earlier investigation on these tribes had revealed that 63 animal species were hunted for their medicinal value and their meat [24]. However, animals and their parts also play important roles for barter and in rituals, religious beliefs, myths and mysticism, the manufacture of goods, decorations and clothing, in stories, song and dance, etc., and in this paper we therefore report that at least 39 animal species among the Tangsa and 38 among the Wancho are associated with tribal traditional knowledge and can be considered of cultural value. This paper will examine if there is a conflict between, on the one hand, the need to keep cultural traditions alive and on the other, to make sure that wild species used for traditional purposes will not become extinct due to the pressure they are under given the roles they play in the local traditions.

## 2. Material and Methods

### 2.1. The Tangsa Tribe

Known for its picturesque hills, Changlang, bounded by the districts of Tinsukia (Assam and Arunachal Pradesh) in the north, Tirap in the west and Myanmar in the southeast, is home to the Tangsa. With an area of 4662 km^2^ the district lies between 26°40’ N–27°40’ N latitudes and 95°11’ E–97°11’ E longitudes and has a majorly Tangsa population of 148,226. Some other tribes, including Singpho, Tutsa, Lisu (Yobin) and Deori also call the district home. Tangsas have a high number of subtribes, namely Muklom, Longchang, Mossang, Jugli, Kimsing, Tikhak, Ronrang, Mungrey, Lungphi, Longri, Havi, Ponthai, Sangwal, Yungkuk, Sakieng and Thamphang. The subtribes reside together and maintain peace and harmony by sharing traditional practices, but they differ slightly in terms of dialect and some attitudes. This study includes information from the Mossang, Muklom, Longchang, Jugli, Tikhak and Kimsing, whose villages were accessible at the time of this study. 

Climatic conditions vary from place to place due to the district’s topography. The altitude ranges from 200 to 4500 m, with lower elevations and the valleys experiencing hot and humid June–August conditions and the settlements in the hills enjoying more moderate weather. January is the coldest month and the average minimum and maximum temperatures throughout the district are 13 °C and 22 °C, respectively. August is the hottest month during which temperatures may occasionally exceed 30 °C. Annual precipitation is 3800–4866 mm with a maximum during June–October. The Tangsa depend about 80% on agriculture for their livelihoods. Shifting cultivation is traditionally practiced, although people have started to adopt wet cultivation technology.

### 2.2. The Wancho Tribe

The Wancho people inhabit the Longding district within a 27°012′ N and 27°132′ N latitude and a 95°16′ E to 95°20′ E longitude, bounded by the Tirap district of Arunachal Pradesh in the east, Nagaland in the west, Assam to the north and Myanmar in the south. The district has a population of around 70,000. The literacy rate of Longding inhabitants is 68.50%. Economically backward with numerous school dropouts and poor healthcare facilities, the Wancho had fought hard for a separate district, which was granted by the Government of Arunachal Pradesh on March 19th, 2012. 

The district is majorly inhabited by the Wancho, who practiced head-hunting until 1991. Nocte and Konyak Naga (also former headhunters) occupy some areas of the district. Owing to the district’s large and diverse geography, Wancho have developed complex social norms, beliefs and practices. Being followers of animism, the society was engrossed by myths, superstitions, tattoo customs and rituals, but since being influenced by Christian missionaries, they have begun to condemn head-hunting and some rituals that accompanied traditional festivals and events. Governed by the council of chieftains in which the King is the Head, traditions of gun-making, woodcarving, bead-making and facial tattooing still exist. Slash-and-burn cultivation known as “*jhum*” is practiced in the mountainous regions. 

### 2.3. Field Survey Descriptions

Field surveys were conducted from May 2015 to March 2016 among the Tangsa and Wancho in their respective districts, i.e., Changlang and Longding in the North-East Indian state of Arunachal Pradesh (26°28′ and 29°30′ N latitude 13 and 90°30′ and 97°30′ E). The region has few roads and many villages are only reachable on foot or at certain times of the year. Ten accessible villages were visited in each of the two tribal areas. The number of households in the Changlang district varied from 30 to 60 per village but was higher with around 100 to 200 per village in the Longding district. Surveys were based on 1–2 h long group discussions and information gathered from semi-structured interviews combined with free conversations at each village headman’s house (i.e., the “Gaon burha”). 

During the interviews, animal photographs were shown to twenty approximately 45- to 70-year old villagers, (the majority being men in keeping with the custom that they are the leaders). Additionally, at least two households inhabited by village elders (aged 80–90: people often do not know their ages) and their families were visited in the two districts. Recommendations by headmen or village elders to visit certain persons were sometimes followed in line with the “snowball method” [25]. Headmen were chosen as initial contacts because of their status, influence and knowledge of their areas’ residents. 

The interviewed people were asked simple questions on whether the animals shown to them in photographs and videos were hunted for food or were involved in rituals, treating diseases, decorating garments, aspects of folklore and anything else deemed important. When animals or their parts were involved, we requested that the specimens be shown to us for identification with illustrated guides [26,27,28]. When this was unsuccessful, photographs of the specimens were later shown to experts or compared with material held in the university collection. Due to the study area’s remoteness and the fact that some animals represented protected species, voucher specimens were not collected. 

Vernacular names of the animals were recorded, but as the locals had limited knowledge of Hindi or English, Assamese as the *lingua franca* was frequently used. Younger people could often communicate with us in English. Since one of us (Salomi Jugli) was a Tangsa, questions could be asked in Tangsa, which facilitated collecting information from members of that tribe. Knowledge of the animals and their uses pass from generation to generation, but now some traditions are declining.

## 3. Results

### 3.1. Animals Associated with Indigenous Hunting Systems of Animals and Commercialization

With a preference for non-domesticated species, the Tangsa and Wancho use a variety of invertebrate as well as vertebrate animals therapeutically and as a source of protein. One exception are vultures (*Gyps* spp.), which are considered taboo by members of both tribes and regarded as dirty and unpalatable. Moreover, there is the fear that they could conceivably have incorporated human remains into their body and they are, therefore, ignored.

All of the tribal communities of North-East India employ various kinds of hunting tools and use different strategies to obtain their prey. Animal traps are constructed using materials like bamboo, tree branches and leaves, but firearms have become increasingly popular. Tribal people may, according to Indian law, possess firearms to defend themselves when attacked by a wild animal, but the Wancho in particular, as our informants stated, use guns more for hunting than for self-defence. 

Hunting and fishing by a group of male villagers are still common Wancho practices. In connection with predictions by experienced hunters for possible successes in hunting, fishing and honey collections, special rituals are performed. These frequently involve an inspection of the inner organs, such as heart, liver, gallbladder and spleen of sacrificed animals. Decisions whether to take a banana, broom or betel leaves along to pack and wrap the anticipated catch in, also depend on the predictions.

### 3.2. Animals Associated with Traditional Uses and Socio-Cultural Aspects

Many similarities between the Tangsa and Wancho exist regarding traditional and cultural uses of animals. For instance, the fur of a bear and the teeth of wild boars may be attached to local headwear by members of both tribes, and while shoulder bands made of goat hair (dyed locally) are usually worn by males during festivals, porcupine quills are the females’ hair accessories in both tribes. Quills also find use in weaving dresses, designed by Tangsa women, while hornbill feathers are attached to head bands of both male and female Wancho but only male Tangsa dancers. Skulls and jaws of animals like deer, buffalo, mithun (*Bos frontalis*) and the beaks of hornbills are used by both tribes to decorate the houses of expert hunters to indicate the latter’s prowess and superiority.

#### 3.2.1. Tangsa Customs

Members of the Tangsa tribe use the feathers of jungle fowl, domesticated hens and roosters as accessories on head bands worn by males and females. The teeth of big cats like *Panthera tigris, Neofelis nebulosa* and *Panthera pardus* are hung on the sheath of local machetes (dao) called ‘*Jang*’ to indicate superiority and strength, while the neighbouring Monpa tribals decorate their dao with the skins and furs of wild goats [5]. Only village heads, local priests and the elderly of a village used to carry machetes with these accessories, but now any rich person may own a decorated machete. The teeth of wild or domesticated boars are used as the trigger in bows (‘*dahkau*’) to dislodge arrows (‘*dahsaan*’). 

The traditional Tangsa drum “*nong*” is played by men during a festival called “*moh-mol*” or, rarely, on other occasions. Anyone, even by mistake, who plays the drum during non-occasions is thought to invite bad luck and receives a hefty fine, which is decided by village elders and imposed on the family of the one who played. Deer hide is the preferred raw material for the local drumhead, but recently monkey, cow, buffalo, or goat skins, e.g., *Capricornis sumatraensis* and *Capra indicus*, may be used when deerskin is unavailable. Dried skins of buffalo, cow and deer are used as carpets; buffalo skins also served as shields called ‘*laak*’. Deer antlers are worn as headgear by men. The tradition to present freshwater fish to relatives and close friends for weddings lives on among some Tangsa subtribes. 

Items considered valuable, possessing special powers or representing a connection with deceased relatives are passed on from generation to generation. However, accessories used by males, e.g., daos, shields, headgear or by females like feathers, beads and armbands are inherited by sons and daughters, respectively. Bows and arrows are male items like drums, although exceptions regarding the latter exist. (Table 1) 

#### 3.2.2. Wancho Customs

Among the Wancho, teeth and nails of bears are worn as ornaments (necklaces) by males. Skins of any monkey species may be used in making baskets and hats for males during festivals (as Monpa tribals do) [5]. Squirrel tails worn by male dancers, who may also wear deer antler earrings, are tied to small baskets attached to their waist. The squirrel tail hanging from the basket around the waist (or attached to the hats) indicates masculinity and is considered beautiful. Mithun (*Bos frontalis*) skin was traditionally used for making defensive shields, as the hide was considered especially tough and even bulletproof. Goat hair, painted red and black with local dyes obtained from a variety of plants, is used on hats and swords worn by male dancers during festivals. 

Beeswax known as ‘*nah*’ helps to fuse rope strands and finds applications in the manufacture of the local muscial instrument known as ‘*patwazah*’. Without beeswax the sound produced by the instrument lacks its characteristic soft resonance that is so loved by the locals. People believe that the wax smoothens rope and bamboo and helps create a more melodious sound. The wax of a bee, known as ‘*Nyahsa*’ is used in connection with ornaments and to attach items made of metal (i.e., mostly copper and sometimes iron), bent into desired shapes, e.g., during the manufacture of guns. 

A tradition of family members and relatives is communal fishing whenever a boy is born. The one who names the child is presented with the day’s catch. Community fishing is also executed during times when crops are collected from the nursery and are separated accordingly. The meat of soft-boiled snakehead fish identified as *Channa* sp. (possibly being *Channa melanostigma*: [29]) is given to infants of both genders just before starting them on solid food at 5–6 months of age. Nowadays this practice is being followed by increasingly fewer mothers (Table 2).

### 3.3. Animals Associated with Indigenous Beliefs, Myth, Rituals and Customs, Cosmologies, Stories and Songs

The notion that diseases are caused by evil spirits, and therefore can be cured only by appeasing the evil spirits by means of an animal sacrifice is still prevalent in North-East India. Domesticated animals like fowl, pigs and dogs are easily available and are sacrificed in the hope to appease the evil spirits. Hoolock gibbons are considered bad omens by the Tangsa and any unexpected death of a family member is blamed on them. If there are unnatural deaths due to an accident or during childbirth, the villagers hunt down a hoolock gibbon and cut its meat into pieces for the relatives of the deceased, but not to be consumed by them but to be thrown away in the jungle, thereby cursing the monkey for having caused the unexpected death (Table 1). 

In Tangsa villages, only the head of a dog or a goat, dried shells of tortoises and honeycomb refuse are frequently seen at the entrance of a house to cast away evil spirits and bad omens. At the onset of labour during childbirth, a dog, later to be sacrificed, may be taken into the forest and tied to a banyan tree. It is believed that this helps the delivery. Similarly, a bunch of monkey hair (any species) is tied to the entrance of the house or attached to the ceiling or beams inside to prevent evil spirits from lodging in the house and giving the family a bad omen, sabotaging its future or causing illness and distress (Table 1).

Pigs and hens often have their livers used in fortune telling and predicting the yield of harvests. Fortune tellers never reveal how they deduce the future from, for example, the liver’s colour, shape and texture. Their final conclusion depends on what their senses tell them with onlookers unable to comprehend how they make the predictions. Liver, gallbladder, heart and spleen used in this haruspex (i.e., interpretations of omens through inspections of sacrificial animals’ entrails) are consumed together with the meat of the sacrificed animal. Buffaloes are only sacrificed during major epidemics and their meat would, of course, be consumed while their skulls would be used for decorations. To ward off evil spirits that attack children and sickly adults, a necklace of frogs’ forelimbs is worn by the weak individual (Table 1). 

Amongst the Wancho, dogs are important and sacrificed, e.g., when a king from one village marries a queen from another. The dog is beheaded with a machete in a single blow, a task usually performed by the king’s eldest brother (signifying the first-born). The queen then takes the first few steps into the in-law’s house by stepping into the dog’s blood on the ground. Upon that the dog’s meat is prepared for consumption by the newly-weds and their relatives. The dog’s significance among the Wancho is also evident from the age-old custom of exchanging dogs as gifts among members with similar surnames. Contrary to the Tangsa, Wancho elders prescribe dog sacrifices also at times of epidemics. All the villagers will touch the sacrificed animal’s blood, as it is thought to possess healing powers and to prevent diseases. The meat will be consumed (Table 2). 

Following community hunting, a killed animal’s head and thighs, considered the most important parts, go to people of high standing while the remainder will be available to all villagers. In connection with an individual’s hunt, the person who shoots the animal is entitled to have the right of the thighs; the one to hit the animal thereafter or to arrive at the spot it was shot, gets the shoulder or hip portion, while the third gets the back. The head is always presented to the village’s king or headman. Members of the tribe believe one should never encounter a python, but if accidentally someone does run into one, the python must be killed by that very person who encountered the snake lest that person be cursed indefinitely. To render a curse ineffective, a priest may perform a variety of rituals (Table 2). 

Orthopterans (e.g., grasshoppers, crickets, etc.) are collected twice: just before the planting of the *Colocasia* (known as “taro”) at the beginning of February and during the Oriah festival in March or April. The ritual, which involves chanting, sacrificing and consuming insects, is performed in order to protect this plant, its edible tuber and other cultivated crops like rice, millet and maize from harmful insects Table 2). 

Traditional songs and stories are preserved and transmitted orally from one generation to another, a process which heavily involves the elderly of the village. Since there are no written records of the stories or the songs and their origins to save them, scientific documentations, videos or audio recordings are required. It should be mentioned, however, that a script for Wancho was recently invented and the first book in Wancho was published by Losu [30]. Songs and stories about animals reflect the importance that the locals attach to species that play roles in their cosmology as a source of food or danger, good or evil. As such, the local folklore can therefore help to shed some light on the various reasons why some species are hunted and consumed while others are killed and not used in any way, or why some are simply ignored.

#### 3.3.1. Stories of the Tangsa

*1)*  *Story 1: The Origin of the Flying Lizard (Junglep)*

Known as ‘*Junglep*’, the name refers to a lizard, whose sound is heard during the months of March and April. During sacrificial rituals (called ‘*Khaatang*’) and head-hunting, female members of the family serve rice beer to male members while the males reciprocate by distributing meat to the females. According to the story of *Junglep*, a teenage orphaned girl, had nobody she could serve rice beer to or receive any meat from in return. The grief of being alone made her cry “*Junghong le, Wahong Le*”- (no elders, father or brothers). She eventually succumbed to her grief and turned into a *Junglep*.

*2)*  *Story 2: Transformation of a Man into a Tiger (Chaah)*

People of the village where this story is being told believe in a “shape-shifting spirit” called “*Namphi*” (or *Aphi*), which can transform itself into a tiger. This is the story of two friends, with one of them disappearing night after night from his bed, making the other suspicious. Getting increasingly worried, the friend decided to find out the reason for his companion’s absences. Searching everywhere for his companion without finding him, he found a container with fresh tiger teeth and claws. He then understood that his friend was a normal human by day and a tiger by night and decided to kill his shape-shifting friend. He put hot charcoal and ashes into the container to destroy the tiger’s teeth and claws, thereby killing the shape-shifter.

Another story about a shape-shifter and a tiger involves a wandering businessman who starts quarrelling with a person of a village he is visiting for some business matter. The person gets furious and warns him that he will teach him a lesson. The businessman on his way back to his own village encounters a tiger. The tiger threatens and thereafter haunts but does not kill the man. The man gets seriously ill after reaching home. After that incident, the villagers conclude that the tiger was not a real animal, but a shape-shifting spirit of the man from the other village with whom he quarrelled.

*3)*  *Story 3: Dog (Heeh) and Pig (Wak) Story: Why Pigs Are Reared and Fed Separately and Dogs Get Just Leftovers*

Once a master had a dog and a pig. Both helped their master in every possible way, but one day the master sent both of them to plough the field. The pig obediently ploughed the field the whole day, but the dog only slept. In the evening, when they were returning home, they had to cross a bridge. The pig having pointed toes could not cross the bridge and had to go down into the water to cross the river while the dog crossed the bridge. Thus, the dog reached home early and told the master that he worked hard the whole day while the pig slept and did nothing. The pig overheard the dog’s lies, confronted him, and told the master the truth. Upon that, the dog got mad and bit the pig, which infuriated the master so that he beat the dog. He then cooked food for the pig but did not bother to prepare any food for the dog. This is why people in the villages still rear pigs and cook their food separately while dogs are fed leftovers of meals served to humans.

*4)*  *Black Bird (Black Drongo) and Rat Story: How the Bird Got Its Beautiful Tail Feathers Shortened*

The black drongo bird used to have very beautiful long and thick tail feathers of which it was very proud. Every day the bird would sit on the highest branch of a bamboo plant and flaunt its tail. The other forest animals loathed this behaviour and decided to teach the bird a lesson. A small bamboo rat decided to take the challenge. When, as usual, the bird sat on the bamboo showing off its tail feathers, the rat climbed up to the bird. Not being noticed, the rat then carefully started biting off the bird’s tail feathers. Thus, the bird’s tail was shortened and to this very day, the black drongo bird has a short tail. To see this boastful braggart taught a lesson made the other animals very happy.

#### 3.3.2. Stories of the Wancho

***1)***  *Story 1: Tiger (Sahnuh) and Cicada (Nyu): How Humans Learnt to Make Fire*

Originally humans and animals lived peacefully together and the tiger was the only one who knew how to make fire but would not share that knowledge. The cicada is considered a mythical creature with eyes on its belly. Once the tiger and the cicada, known by the Wancho as “*nyu*”, were keeping company. For some reason, the tiger had to make fire and, not wanting to disclose how he did it, asked the cicada to close its eyes. The cicada complied and started to wrap its arms and legs around its belly to cover its eyes. Not realizing that cicadas have eyes on their bellies, the tiger scolded the cicada and demanded that it cover its head, thinking that the cicada’s eyes must be there. The cicada, however, watching the tiger make fire learnt the technique and then conveyed that knowledge to the humans. That was how the world learnt how to make fire (*wuju*).

***2)***  *Story 2: Owl (Akhuh): The Day and Night Decider and the Origin of the Owl’s Flat Head*

An argument once started between nocturnal and diurnal animals. Diurnal animals claimed there was only daylight throughout a day and darkness. The nocturnal animals took the opposite view, saying there was only darkness in a day. Then came the owl and announced that both light (day) and dark (night) were in a day. The animals agreed that the owl should become the day and night decider. However, they were annoyed with the owl because had it arrived earlier, they needn’t have had an argument. They decided upon a punishment and agreed that hitting the owl on the head and singing “why did you not come earlier to teach us, so that our fight would not have happened?” (“*Akhuh thele thele amih saman ngui hait*”) would be the appropriate punishment. The beatings on the head of the owl then the flattened this bird’s head.

***3)***  *Story 3: Crab (Saan) and Frog (Luk): How the Crab Got Its Colour and the Frog Lost Its Backbone*

The frog and the crab were ploughing the field. The crab brought boiled colocasia (= taro) for lunch. The colocasia became cold by noon, and so the crab decided to warm it up by putting it on the fire. When the crab went near the fire to retrieve it, the crab’s body turned red in colour due to the heat. Seeing the crab becoming red, the frog could not stop laughing and laughed so hard that he bent his backbone. Since then, crabs have a red body and frogs lack a straight backbone.

***4)***  *Story 4: Mongoose and Blackbird: How the Bird Got Its Tail Feathers Thinned and the Mongoose Got Sharp Teeth and a Long Mouth*

Using its mouth, a mongoose once took hold of a blackbird’s tail. The bird, in order to get rid of the mongoose, started flying upward. However, the adamant mongoose did not let go and was dragged up into the air by the bird. The struggling of the bird eventually made the mongoose lose its hold and therefore, to this day, the bird’s tail is thin along the base and normal at the tip, while the mongoose’s teeth in its elongated mouth are sharp and pointed.

***5)***  *Story 5: The Origin of the Monkey*

Ages ago, there lived a couple who adopted an orphan as their son. They asked their son to take care of their paddy field in the jungle. Being alone, the son enjoyed his freedom and played and slept but did not bother looking after the field. One day, the parents conveyed the message to return to their son through a neighbour, who had his own paddy field nearby. The neighbour, however, told the son that his parents want him to stay in the jungle and never to return. So the parents waited for their son to arrive in vain. They conveyed the same message again through their neighbour and also sent their son some food. Yet again, the neighbour misled the son into thinking his parents were waiting to beat and kill him. Consequently, the son decided not to return home. The father then decided to go to the field with some food himself to make his son come back. On reaching the field, he called his son, but the son got so frightened that he ran deeper into the jungle. His father was shocked and followed him. He requested him to come to him and have some food, but the son still thought his father had come to beat him for not taking care of the field. His father showed him the special meal he had cooked, but the son climbed a tree and shouted to his father that he had enough to eat what he liked best, namely the young leaves of the bamboo. The boy then disappeared into the jungle to turn into a monkey and never to return.

*Song 1: Honey Bees: the messengers of love*

“Nahkat peele jangpong thaiba

Zole taahpo kah ho le lah hai”

***Narration***: Whenever you are sitting alone and a honeybee flies buzzing around you, you must never kill it, as it is believed to be the spirit of a loved one who is thinking of you.

*Song 2: Cicada*

“Sacchi sahpu ngu nu din sammai”

***Narration****:* This line says about the cicada that, although its stomach is always empty, it still has a beautiful and sweet singing voice. In the early days when traditional methods were used for cultivating paddy, the yield was low and would often not even last until the next harvest. Just before the harvest season, when people had nothing in their stores to eat, it was the time when cicadas began to sing. The starving villagers took inspiration from the cicada’s singing, for even on an empty stomach it never ceased to sing. People likewise thought they should not stop working if their stomachs were empty. 

## 4. Discussion

### 4.1. Traditional Knowledge: Looking Back on the Way Forward

A study like this based on a limited number of interviews with a fraction of the population can only “scratch the surface” of what would constitute the totality of tribal lore regarding animals that the Wancho and Tangsa encounter in their respective areas. Given, however, the speed with which traditional beliefs and age-old practices disappear, any bit of information still available is precious and worth reporting. 

There are, of course, efforts by community leaders, scholars and the government to slow down this erosion and gradual disappearance of tribal traditions, but if such efforts do prove successful, new problems may arise. As the tribal population increases and members of the tribes continue to follow their traditional practices related to wild animals, species already vulnerable and threatened by extinction may not survive into the future. It is this dilemma that anthropologists, animal conservationists, law-makers and policing bodies have to grapple with.

The general concern regarding traditional uses of animals is therefore twofold. If we do not have in place encouragements and perhaps even rewards to retain local traditions, we will lose them and once lost, they would be almost impossible to revive. On the other hand, cultural purposes revolving around the exploitation of certain animals and the impact such practices can have, need to be addressed. This study has revealed only some instances that would require judicial intervention as there was no immediate threat to most of the animals (other than the large carnivores, the hoolock gibbon, slow loris and pangolin) used by the tribes. In fact, it is in the interest of the tribal people that their ritually important species continue to be available (with the exception of a few like the pythons which are loathed and considered dangerous to individuals and the community: but see the next paragraph). 

### 4.2. Living in Harmony with the Animals: An Achievable Goal?

The emphasis on maintaining their rituals and customs, their beliefs, myths, etc., could be a guarantor for the tribals’ protection of various species, working against needless animal killings. In this context, traps constructed by using materials like bamboo, tree branches, leaves, and stones as described in [31,32] are far superior to the indiscriminate use of firearms in order to obtain terrestrial species or the use of fine nets of polyamide fibres like nylon to snare aquatic or aerial organisms. However, traditional beliefs can also be helpful to protect certain species. To mention a few examples: eagles, considered endangered, are believed by the Tangsa to possess the spirits of dead people and are never harmed. Pregnant and lactating Tangsa females avoid pythons and tortoises lest their babies stick out their tongue, be incessantly salivating and requiring prolonged feeding on breast milk to avoid allergies or skin problems. It is also believed that consumption of tortoise meat by pregnant females may cause their babies to walk awkwardly and although the shells of tortoises are sometimes used to ward off evil from a house, tortoise populations are not in danger of being overexploited by the locals. Regarding crabs, the Tangsa are advised to consume them seldomly and then only in small quantities, as the consumption could lead to malaria. Some Tangsa believe that to hunt hornbills unless seen in pairs is a sin and that three eggs should never be collected together. Hornbills are said to lay only three eggs; one each for the heavens, the earth and a hatchling. 

The Wancho also consider eagles to possess the spirits of the dead and their consumption is taboo. Crows are avoided as they are considered dirt sweepers and vultures, including the critically endangered *Gyps indicus*, are not hunted, but mainly ignored because they are seen as unclean. Moles and slow lorises are animals whose reputations are entirely negative and even the sight of them is a bad omen. Although they may sometimes be killed, more often they are simply avoided as it is an effort to hunt them down. Cobras are believed to possess the souls of people and if killed, they will haunt and ultimately destroy the person who harmed the snake. The Wancho avoid urinating or defaecating onto a termite’s mound, locally called ‘*hahpho*’, as it could cause problems like swelling of the genitals and buttocks. One should not touch the mound and has to ensure that the mud from the nest is not exposed to the mud used for making the fireplace. Although there is definitely no need to protect the termites, beliefs such as these (and almost certainly many unrecorded more) were strictly adhered to in the past but have undergone some relaxation. The present generation, for example, does collect and relish termites, especially during their swarming phase. 

Bee honey and brood are collected systematically. When a hive is found, the village king is informed and family members and some villagers accompany the person who located the hive to retrieve it. However, they need to listen to some predictions first to help them estimate the size of the hive, the quantity of the honey and its brood. As the brood and quantity of the honey matter to safeguard the resource, any attempts of extraction are abandoned if the yield is predicted to be small. The proficient utilization of honeybee products also differs. Beeswax has several purposes for the Wancho but not the Tangsa. Insects generally, and crickets and grasshoppers in particular, play a bigger cultural role for the Wancho than they do for the Tangsa [33] and this is reflected in some of their folktales and stories.

### 4.3. What Do the Differences Mean to a Biodiversity under Threat?

Several aspects regarding cultural practices and traditions unite the two tribes, but obviously some differences exist, especially in connection with the killing of species belonging to the cat family (i.e., tiger, leopard, etc.). Where, on the one hand, some Tangsa participate in merry-making and feasting following such kills, the Wancho tribals consider such killings unfortunate and become seriously concerned, making the villagers perform various rituals after such killings. Another difference concerns the hoolock gibbons. For the Tangsa, even to set eyes on one is thought to have serious consequences for the person who saw the gibbon. For the latter, already an endangered species, the consequences could be even worse as it may be killed to avert any harm coming to the person who saw it. The Wancho know no such repercussion regarding the hoolock gibbon, but kill the slow loris, which, however, are of little interest to the Tangsa. Obviously, there are threats to some already vulnerable species and the pangolin is one of those species under particular pressures: it is used for trade and barter by both tribes, and moreover for food by the Tangsa. 

To stop the needless killings, one needs to understand the reasons why the animals are killed. If it is for food, alternatives could possibly be provided by domestic species. Opportunities to earn money other than by selling wildlife should reduce the pressure on the traded animal species. Animal parts, and this includes feathers, used for decoration and adornment can be replaced by equally beautiful artificial objects, and when it comes to the use of species in magic and rituals, the best option is to make people understand that they will lose their ritually important species if they don’t protect them and make sure there are always sufficient younger individuals to replace those removed from the population. Tribal people need to understand that biodiversity matters; that is, that it helps to stabilize the environment they call “home” and the younger generation can teach the older one. 

Luckily, local leaders and elders are still widely respected individuals and to empower such local leaders by letting them feel that they are the guardians of their region’s flora and fauna and thereby have a responsibility to make sure that unnecessary killings do not take place, can be very helpful. This means, of course, that the leaders and elders themselves have to understand that some of the older beliefs like the hoolock gibbons’ bad omens are no longer acceptable and that the harmless pangolin (and the income derived from the sale of its scales) will disappear if there are no restrictions to curb its exploitation. Laws and regulations have to be put in place, but law enforcement is weak in the remote areas, which is why those that are influential in the community need to be convinced that some of the older beliefs should not be upheld any longer and that alternatives to the use of wildlife in rituals can be found: some domestic species could, for example, be used and some like dogs and chickens are indeed already used. 

The examples given in the previous section in connection with the attitudes towards certain animal species show differences between the tribes, especially regarding the endangered cat species and hoolock gibbons. Although climate and environmental features as well as faunal composition seem similar between the two regions, there could be differences regarding the abundance and occurrence of certain species. It is, however, also possible that cultural dissimilarities and attitudes have evolved between the tribes to deliberately separate the two populations from each other in ways that food and other taboos are distinct between neigbouring ethnic groups [34]. Contacts with missionaries and western ideas may also have varied between the tribes and led to separate attitudes towards species in their respective regions. 

Although both tribes rely on predictions (made by respected local experts, who frequently use animals or animal parts) before going to collect wildlife or go hunting, the practice has now almost disappeared among the Tangsa, largely due to the influence of Christianity, but still operates among the Wancho. Likewise, the practice of “community hunting”: it is nowadays only still common among the Wanchos. The Tangsa may very occasionally go “community hunting”, but group hunting (involving a few people) and individual hunting are nowadays more common activities and possibly less damaging to the wildlife than if whole crowds of people were going on a hunt all together. It is encouraging that the stories retold in this essay do not generally describe hunts or depict the animals as foes and enemies of humans but greet them as partners. The stories may not be identical but they are a reflection of how the members of the two tribes interpret Nature and her creatures. The connection with the animals of the region is something that is shared by the stories of both tribes and this demonstrates that the species mentioned in the stories are important representatives of the wildlife of the region—and hopefully will remain to be so in the future as well.

The Government is lending a helping hand to promote and educate tribal people of Arunachal Pradesh to preserve the cultural uses of their animals without losing the region’s biodiversity or threatened and vulnerable species. Tribal leaders do generally support the government’s efforts, but their powers to influence and/or control the behaviour of their people will inevitably steadily get weaker. Ultimately, the tribal people of the region themselves need to find a compromise between, on the one hand, accepting an increasingly more modern “Western” lifestyle and on the other, retaining their local customs that define their tribes’ respective cultural uniqueness.

## Figures and Tables

**Table 1:** Traditional knowledge associated with the Tangsa tribe.

**1. Mammalia**					
**Sl.**	**Scientific Name**	**Common Name**	**Local Name**	**Parts Used**	**Purpose and Traditional Use**
1	*Ursus thibetanus,* *Melursus ursinus*	Asiatic black bear, Sloth bear	Chabbaang	Hair, gall bladder	*Decoration:* Used for traditional hats and for making shoulder bands worn by men during festivals. *Utilty:* Knives used for cutting bamboo for house construction are rubbed with a piece of dried bear gall bladder, soaked in water. This helps to avoid the bamboo being attacked/damaged by pests.
2	*1) Macaca assamensis,* *2) Macaca mulatta,* *3) Trachypithichus pileatus*	1) Assamese macaque, 2) Rhesus macaque, 3) Capped langur	1) Wii till 2) Woi 3) Raq	Hair	*Magic*: Hair used on traditional hat worn by male and for preventing evil spirit causing bad omen.
3	*Hoolock leuconedys*	Hoolock gibbon	Thukbai		*Magic*: Gibbons cause bad omens (accidents, unnatural deaths). Whenever an incident happens, villagers go and hunt a gibbon, kill it, cut its body into pieces and throw them away. It is believed that the spirit of the dead person has been taken away by the monkey.
4	*Manis pentadactyla*	Chinese Pangolin	Bitsai	Scale	*Magic*: Believed that out of its many scales some would consist of imaginary pictures featuring deer, maidens, temples (used for worshiping) etc. Such scales are considered highly valuable and kept as treasures. The rest are sold. *Food*: The animal is killed for meat and the scales are traded to neighbour states and countries like Nagaland, Assam and Burma.
5	*Vulpes bengalensis*	Fox	Makakoi	Flesh, Blood	*Bad food*: Only few people consume its flesh, as some consider it as unclean as foxes feed on carcass. *Magic*: If blood is consumed no black magic can harm the consumer.
6	*1) Canis lupusfam.* *2) Capra hircus*	1) Dog 2) Goat	1) Heeh, 2) Kekai	Head	*Magic*: To appease a bad omen or evil spirit the head of dog or goat is severed and kept in front of the house; the body is buried near or in front of the owner’s house’s stairs; an event called “*Khulek*”.
7	*Canis lupus fam.*	Dog	Heeh		*Sacrifice:* During pregnancy or before birth or for a healthy delivery a dog is taken to the forest and tied only to a Bunyan tree (*Min chung*) and sacrificed with continuous chanting.
8	*Capra hircus*	Goat	Kekai	Hair from beard	*Decoration*: Hair from the goat’s chin (most preferred) is used for making shoulder bands worn by men during festivals.
9	*Sus scrofa* *domesticus*	Domestic pig	Wak	Liver	*Magic*: For performing predictions; fortune telling before harvest and cultivation.
10	*Sus scrofa*	Wild boar	Wakngi	Teeth	*Decoration*: Teeth used for the men’s traditional cap for decoration and as a symbol of male power and strength.
11	*Hystrix* sp.	Porcupine	Wihaang	Spines	*Decoration*: Spines are worn by women on their head for beautification and as hair accessories during festivals. *Magic*: Used for performing black magic by some tribals. *Utility*: Spines are used for weaving local costumes, mainly for putting designs on them.
12	*Cynopterus sphinx*	Bat	Phaksak		*Poor food*: Only consumed by adults (mostly males) with most of the population believing its taste to be unpleasant and linked to bad omens.
13	*Nycticebus* spp.	Slow Loris	Rangchuwi		*Magic*: In the past, it was believed that seeing this animal itself causes a bad omen, following the owner when leaving an earlier home and moving to the new house.
14	*1) Panthera tigris,* 2) *Neofelis nebulosa,* *3) Pantherapardus*	Cat family: 1) Tiger 2) Clouded leopard 3) Common leopard	1) Chaah 2) Pulkhu Chaah	Teeth	*Exalted species*: Some subtribes (e.g., like Jugli) do not eat bornbills and tigers as they consider them to be “Kings” (royal). It is believed that the tiger’s soul or spirit is related to humans and a person would soon die too. The cause of the death of the tiger, any marks on its body when hurt, would be seen as the exact place on the dead person. *Magic*: When someone kills any cat family member, a feast for all villagers with rituals to prevent a bad omen is given by sub-tribes Muklom, Tikhak and Longchang, as it is believed that the soul of a *Shamma* (priest) dwells in the tiger. Teeth (canines) are presented as a gift to the maternal uncle. The incisors are presented to village heads/elders like Gaon bura. Bones and skin are sold at the market. *Decoration*: Teeth of all cat family members are attached to swords of important elders, village heads like *Gaon bura*, village judges like ‘*Walang*’ etc. Teeth signify male power and symbolize good luck.
15	*Bos bubalus*	Buffalo	Loi	Head, Skin	*Sacrifice*: Buffaloes are sacrificed whenever there is an epidemic (spread of disease or any evil spirit) *Decoration*: The head is used for decorative purposes. *Utility*: The dried skin is used for shield called ‘*Laak*’, but also for making drums played at festivals or for making carpets to sit on.
16	1)*Cervus uni* 2) *Axis porcinus*	1) Sambar deer 2) Hog deer	Chok/Khihoi Nalang	Skin	*Utility*: The dried skin is used for making drums (the thinner the better) and also for making carpets to sit on. Deer skins are the most preferred for making drums.
17	*Bos indicus*	Cow	Maan	Skin	*Utility*: The dried skin is used for making drums and carpets.
18	*Sciurus* sp.	Squirrel	Chanchaang	Tail	*Decoration & utility*: Dried tails are used for decorative purposes and for making key chains/holders.
**2. Aves**					
**Sl.**	**Scientific Name**	**Common Name**	**Local Name**	**Parts Used**	**Purpose and Traditional Use**
1	*Hornbills:* *1) Buceros bicornis* *2) Aceros nepalensis* *3) A. undulates* *4) Anthracoceros albirostris*	1) Great hornbill 2) Necked hornbill 3) Weathered hornbill 4) Pied hornbill	1) Wuraang 2) Wujung 3) Wungip 4) Wukengkap	Feathers (mostly tail), beak	*Decoration*: Tail feathers (*alap*) from male hornbills (especially the Great Hornbill) are considered most beautiful (with variable colours); used on hats worn by the first dancer who leads the dance troop (*Lamshal waa*) during festivals. However nowadays all male dancers wear it. Beaks are used for decorative purpose. *Magic:* Hornbills, considered “Kings” (royal), are usually not eaten. People feared to bring them home as they were considered to possess ill-causing spirits (the “evil spirit” called *“Wuraang thang”)* causing bad omens and deadly diseases. Moreover, it was believed that when a sick hornbill was killed, the person who killed or touched it would infected with the disease. *Magic:* Few subtribes hunted them, but then only when seen in pairs; it was believed to be a sin to kill a bird that was single. If only one was seen, it meant it was a male and a female was with the nest and the young; thus males must not be killed to keep female’s and young’s company. Only 3 eggs are were thought to be laid: one for Earth, one for Heaven (God), one for the young.
2	*Spilornis cheela*	Crested serpent eagle	Laang		*Magic*: Not edible, as considered to possess the spirit of a dead person. (If a person dies a few days later vultures are seen gliding in the sky and the eagle is believed to be the dead person’s spirit (*jakhang*). Family members place some food and sprinkle water outside the main entrance of the house, as an offering to the spirit). Few sub-tribes consume it, but then only older males do.
3	*Gallus domsticus*	Domestic chicken	Wuu	Liver	*Magic*: Used during performing rituals to bring back a lost spirit considered to be the spirit taken away by a ghost. *Magic*: For fortune telling before harvest and cultivation.
4	*Polyplectron bicalcaratum*	Grey peacock pheasant	Wupoi	Wings, feathers	*Food & Decoration*: Killed for food, but wings and feathers used for decoration due to their colourful appearance.
5	*Gyps* spp.	Vultures		Not used	*Taboo:* Unpalatable and dirty
**3. Reptilia**					
**Sl.**	**Scientific Name**	**Common Name**	**Local Name**	**Parts Used**	**Purpose and Traditional Use**
1	*1) Python reticulatus* *2) Python molurus*	1) Reticulated Python 2) Indian python	Paujung	Skin	*Food & Magic*: The flesh must be consumed either boiled or fried. When roasted, it is believed that the skin colour of the person who eats it, would change into snakeskin with time and age. *Taboo*: Not consumed by pregnant and lactating women as it is believed that the child feeding on breast milk would develop an allergy or skin problem. In case of pregnant women, it is thought that the child would be born with their tongue sticking out, constantly salivating and drooling. *Decoration*: The dried skin is used for decorative purpose.
2	*Varanus* sp.	Monitor lizard	Paupot		*Magic*: It is feared that when bitten, a person dies because of the poison. However, there is the belief that the person might survive if s/he (in disguise) reaches the river before the lizard as it is thought the lizard goes to the river after it has bitten a person. If the lizard reaches the water (river) first, the person would die.
3	*Testudo* sp.	Tortoise	Kongsharang	Shell	*Taboo*: Pregnant women do not eat this reptile fearing their babies might never walk properly or may experience a delay in walking. *Magic*: The dried shell is hung outside the entrance door of the house in order to drive away any evil spirit or bad omens.
**4. Amphibia**					
**Sl.**	**Scientific Name**	**Common Name**	**Local Name**	**Parts Used**	**Purpose and Traditional Use**
1	*Unknown* sp.	Frog	Likkai	Forelimb	*Taboo*: The forelimbs of a frog that are mostly found during late August (they come in groups) are used in the form of a necklace to protect both adults and children from evil spirits. *Magic*: Also believed that when eaten, the man who consumes the frog’s forelimbs will not die quickly (as frogs come in groups).
2	*Bufo* sp.	Toad	Lugmaanchai		*Taboo*: Toads are not consumed as they are considered to be poisonous and when eaten the person dies instantly. Also it is noted that it is not even eaten by other animals.
**5. Pisces**					
**Sl.**	**Scientific Name**	**Common Name**	**Local Name**	**Parts Used**	**Purpose and Traditional Use**
1	*Labeo* sp.	Cyprinid	Ngah		*Social purpose*: Common fresh water fishes are presented as a custom to invite relatives and close friends to special occasions like marriage by the Lungchang sub-tribes.
**6. Insecta**					
**Sl.**	**Scientific Name**	**Common Name**	**Local Name**	**Parts Used**	**Purpose and Traditional Use**
1	unidentified sp.	Cicada	Wajong		*Utility:* This insect with its first sound announces the beginning of the sowing of rice. *Magic* & Health: In the past some subtribes believed that consuming this insect could cause headache. *Magic & Health*: The insect’s sound is believed by some to be a sign of the start of sickness/fever, so that the villagers throw some plant leaves backward as a belief to avoid sickness/fever.
2	Mantodea	Praying Mantis	Rawehpaanpah		*Food & Health*: Nymphs/eggs but not adults are eaten because the latter are believed to possess worms inside their stomach.
3	*Odontotermes* sp.	Termite	Phinphoi		*Food & Health*: Only the nymphs are consumed. Adults when eaten are linked with a person’s swollen stomach and death.
6	*1) Apis cerana* *2) Apis mellifera* *3) Apis dorsata* *4) Apis florea* *5) Apis andreniformis*	Honey bees	1) Nyahkaai 2) Nyahkhing 3) Nyahkaan 4) Nyahbi 5) Minmoi/Tangu	Honeycomb	*Magic*: The empty honeycombs of any honey-producing bees are hung on the entrance door with a belief to cast away evil spirits.
7	*Xylocopa* sp.	Carpenter bee	Bintin	Sting	*Taboo & avoided*: This bee is rare and avoided because its sting causes diseases like swelling of skin, wounds, pus, etc.
**7. Crustacea: Malacostraca**					
**Sl.**	**Scientific Name**	**Common Name**	**Local Name**	**Parts Used**	**Traditional Knowledge**
1	*Maydelliathelphusa* *lugubris*	Freshwater crab	Khaan		*Taboo & health*: It is believed that crab food can cause malaria. Crabs are therefore completely avoided by some subtribes.

**Table 2:** Traditional knowledge associated with the Wancho tribe.

**1. Mammalia**					
**Sl.**	**Scientific Name**	**Common Name**	**Local Name**	**Parts Used**	**Purpose and Traditional Use**
1	*Ursus thibetanus,* *Melursus ursinus*	Asiatic Black bear, Sloth bear	Chapnu	Hair, tooth, nails	*Utilty*: The fur is used for making traditional hats worn by male during festivals. *Decoration*: Teeth and nails used as ornament by males. *Gift & trophy*: One half portion of the hair (mainly from the head region that covers the ears) is presented to the king and the other half is kept by the owner of the slain bear.
2	*1) Macaca assamensis,* *2) Macaca mulatta,* *3) Trachypithichus pileatus*	1) Assamese macaque 2) Rhesus macaque 3) Capped langur	Mainak	Skin	*Utilty*: The skin is used for making caps and baskets that are worn during dancing by the males during festivals.
3	*Manis pentadactyla*	Pangolin	Hahbut		*Trade & barter*: Scales collected from a pangolin are sold to residents of the neighbouring states and countries.
*4*	*Canis lupus familiaris*	Dog	He		*Social purpose*: Dogs are exchanged as gifts, to pay fines etc. but only within people sharing the same surname or subtribe. *Food & health*: Earlier eaten only by lower caste people, but presently preferred by all with the meat being considered medicinal. *Magic & health*: A dog is sacrificed by the oldest man of the community in the presence of all the villagers during an outbreak of an epidemic like measles, small pox, diarrhoea etc. The fresh blood is then touched by the villagers, (both sick and healthy), as it is believed that by doing this they would be healed from the diseases. *Ritual food*: Marriages of kings and queens from different villages are consummated by sacrificing a dog. The sacrifice is done by the king’s eldest brother (signifying the first-born son) by beheading it in a single blow. The newly married queen enters her in-laws’ house by stepping over the spilled blood on the ground.
5	*Hystrix* sp.	Porcupine	Adi/Azi	Spines	*Decoration*: Spines used by women as local earring and by ladies as hair accessories during festivals. *Utility*: Used as forks to eat meat during festivals.
6	*Nycticebus* spp.	Slow Loris	Awai		*Magic*: Not eaten; hunted and killed if sighted, as it is believed to cause bad omens.
7	All Cat family 1) *Panthera tigris* 2) *Neofelis nebulosa* 3) *Panthera pardus*	1) Tiger, 2) Clouded leopard 3) Common leopard	Chahnu		*Taboo:* Not consumed as it is seen to be an incarnation of a King’s soul. Seeing tigers is considered rare for the same reason. Killing of any of the cat family is a serious issue among the villagers (they do not want to kill but have to for safety). Young and old alike would weave small local baskets and carry them to the forest to seek forgiveness from the gods of the Kings (as the great cats are the strongest of all animals in the forest) by singing and dancing. The whole village are considered to be guilty of the crime of killing the animal, which is why everyone participates in the ritual.
8	*Bos bubalus*	Buffalo	Loi	Head/Skull	*Decoration*: For decorative purposes and to exhibit superiority.
9	*Sciurus* sp.	Squirrel	Heeh	Tail	*Decoration*: Used for decorative purpose. On the first day of the Oriya festival, male dancers wear the squirrel tail tied to a hat or local basket, worn around the waist. Smaller squirrel tails are tied to children’s baskets too.
10	*1) Cervus uni* *2) Axis porcinus*	1) Sambar deer 2) Hog deer	Maikhi/Chok	Antlers	*Decoration*: Antlers used as decorations and adornment. Earrings made from antlers worn by males as ornamets. *Social:* The first person to accurately fire the bullet gets the right thigh of the animal. The 2nd person to hit or arrive at the spot, gets the shoulder portion; followed by third with the back portion. The head is always presented to the king of the village (all this is followed only during community hunting and not in individual hunting).
11	*Talpa* sp.	Mole	Thupha		Sighting this animal is believed to be a bad omen and leads to the abandonment of executing further plans or work.
12	*Sus scrofa*	Wild Boar	Myla	Teeth	- The teeth are fixed to hats and worn by males during festivals. - Teeth are used for decorative purposes.
13	*Bos frontalis*	Mithun	Ngaa	- Head/Skull - Skin	- The head/skull is hung outside the house for decoration and as a symbol of superiority/strength. - After drying the skin is used as a shield and for defence during fights with the enemy; it is even used as a bulletproof shield.
16	*Capra hircus*	Goat	Zon	Hair	- The hair is painted red and black using locally made dyes. This painted hair is used as shoulder bands and on hats worn by males. Also tied to swords used for dancing during festivals. - Earlier, members of the high-class section of the tribe did not eat goat, but now they do.
**2. Aves**					
**Sl.**	**Scientific Name**	**Common Name**	**Local Name**	**Parts Used**	**Traditional Knowledge**
1	*Hornbills:* *1) Buceros bicornis* *2) Aceros nepalensis* *3) A. undulates* *4) Anthracoceros albirostris*	1) Great hornbill 2) Necked hornbill 3) Weathered hornbill 4) Pied hornbill	Ozang	Feathers (mostly tail), Beak	- The feathers are used as decoration in traditional hats, both by male and female dancers during festivals. The tail feathers of the great hornbill are more favoured than others. - The beak is also used for decorative purposes.
2	*Spilornis cheela*	Crested serpent eagle	Ola		This bird is considered to possess the spirit of a dead person and s therefore protected and neither hunted nor consumed.
3	*Bubo nipalensis,* *B. bubo*	Owl	Akhuh		- Consumed only by the elderly people. - Considered the decider of day and night.
4	*Dicrurus paradiseus*	Greater racket-tailed drongo	Waah	Feathers	- The feathers are used in traditional hats - Not edible. The bird chirps during dusk and dawn so people believe it to be the morning and night announcer. The chirps are taken as an indication of time to return from the fields in the evening, as it would get dark soon.
5	*Dicrurus macrocercus*	black drongo	Jajoi		It is believed this bird informs the people of the presence of fish in the stream and alerts them whether or not it’s worthwhile to go fishing.
6	*Corvus* *splendens*	Crow	Okha		Not consumed as it is considered a sweeper/ scavenger and thus dirty.
**3. Reptilia**					
**Sl.**	**Scientific Name**	**Common Name**	**Local Name**	**Parts Used**	**Traditional Knowledge**
1	*1) Python reticulatus* *2) Python molurus*	1) Reticulated Python 2) Indian python	Punu		- Hunters should not have eaten until a ritual is performed by a local priest, which is done by removing and using the horn-like outgrowth in the abdomen of the snake. The ritual is concluded by hanging the head of the snake outside the house of the priest (and let it wither away with time). The consequence of not performing the ritual is believed to lead to a cursed life of the hunter. - The snake is killed upon sight or the curse is believed to double or cause bad omens. - The horn-shaped outgrowth is believed to be poisonous and the reason for the withering away of the canopy over which the snake crawled. - Sighting a python could lead to the death of the person, and therefore the python must be killed and cut in two and thrown away to survive the sighting. It is then followed with rituals and feasting.
2	*Naja* sp.	Cobra	Pucham		The soul of people is believed to dwell in this snake and killing it would welcome curses and an eventual death of the person.
**4. Amphibia**					
**Sl.**	**Scientific Name**	**Common Name**	**Local Name**	**Parts Used**	**Traditional Knowledge**
1	Anura (Hylidae)	Green Frog	Luk	Whole Body	Eaten before performing rituals during festival.
**5. Pisces**					
**Sl.**	**Scientific Name**	**Common Name**	**Local Name**	**Parts Used**	**Traditional Knowledge**
1	*Labeo* sp.	Cyprinid	Nyah		- When a boy child is born, the family members and relatives (mostly males) go fishing. The person who names the child gets the maximum share of the caught fish. - Fishing is also done during the time of collection and separation of rice samplings from the nursery.
2	*Channa* sp.	Snakehead fish	Nyah		This fish is fed to infants just before starting the consumption of solid food.
**6. Insecta**					
**Sl.**	**Scientific Name**	**Common Name**	**Local Name**	**Parts Used**	**Traditional Knowledge**
1	*Odontotermes* sp.	Termite	Khunkhah	Termite mound	- Urinating or excreting on the termite mound (*Hahpho*) is believed to cause swelling of the private parts and buttocks. - Contacting the mud from the mound should be avoided and should never be used to build a fireplace in the house.
2	*1) Apis cerana* *2) Apis mellifera* *3) Apis dorsata* *4) Apis florea* *5) Apis andreniformis*	Honey bee	1) Nyaakat 2) Nyaakat 3) Nyaakat 4) Nyahsa 5) Nyahsa	Bee wax	- The presence of a beehive should be informed to the king and then the family, relatives and the villagers. A local prediction is performed using leaves (e.g., banana, betel leaf etc.) before collecting the honey to know the yield (size of honeycomb and quantity of honey and brood inside). Extraction is not performed if the yield is predicted to be low. - Messenger of love. The bee is believed to be the spirit of a lover and its buzzing conveying love. - Wax is used as a coat by rubbing it on local musical instruments, especially the mouth instrument called ‘*Patwazah*’ made of bamboo and rope to produce a better sound from the instrument. - Beeswax is used for joining copper materials and some iron materials too, mostly to shape the barrel of a gun to a desired curvature, but also for making ornaments.
3	Orthoptera	Any grasshoppers and crickets	Okuk		- Freshly caught from rice fields used in rituals: e.g., during Oriya festival and while planting *Colocasia* (taro) eaten in the belief of protecting the plant from the insect and for the healthy growth of the plant. - The insects are collected from the field and the rituals are performed by a priest to ensure healthy cultivation of rice, millets, colocasia etc.
